# Active Brownian motion of strongly coupled charged grains driven by laser radiation in plasma

**DOI:** 10.1038/s41598-022-12354-7

**Published:** 2022-05-21

**Authors:** Oleg F. Petrov, Konstantin B. Statsenko, Mikhail M. Vasiliev

**Affiliations:** Joint Institute for High Temperatures, Russian Academy of Sciences, Moscow, 125412 Russia

**Keywords:** Plasma physics, Colloids

## Abstract

The systems of active Brownian grains can be considered as open systems, in which there is an exchange of energy and matter with the environment. The collective phenomena of active Brownian grains can demonstrate analogies with ordinary phase transitions. We study the active Brownian motion of light-absorbing and strongly interacting grains far from equilibrium suspended in gas discharge under laser irradiation when the nature and intensity of the active motion depend on the effect of radiation. Active Brownian motion is caused by photophoresis, i.e., absorption of laser radiation at the metal-coated surface of the grain creates radiometric force, which in turn drives the grains. We experimentally observed the active Brownian motion of charged grains in the transition of the grain monolayer from the solid to liquid state. An analysis of the character of motion, including the mean-square and linear displacement and persistence length at various values of the randomization (coupling parameter) of the grain structure, was presented.

## Introduction

Considerable recent attention has been focused on active Brownian motion^1^. Unlike passive Brownian grains^2^, which are in thermal equilibrium with the environment, active Brownian grains can convert energy from the environment into the kinetic energy of their motion, as a result of which they find themselves far from equilibrium with the environment^3–6^.

The systems of active Brownian grains can be considered open systems, in which there is an exchange of energy and matter with the environment. Open systems are called dissipative structures if the scattering of energy coming from outside maintains a stationary ordered structure with an entropy less than the equilibrium structure^7,8^. A change (increase) in the flow of entropy into the environment in dissipative structures can lead to their self-organization and evolution^8–11^.

In connection with the above, the study of ensembles of interacting active grains^1^ is fundamentally interesting and important from the point of view of potential applications, for instance, when designing nonliving active Brownian systems^12,13^. The collective phenomena of active Brownian grains can demonstrate analogies with ordinary phase transitions. Thus, it was shown in the work^14^ that for a model of a system of active grains under certain conditions, two-stage melting from solid to hexatic to liquid phase is retained even far from equilibrium. An interdisciplinary study of an active substance allows one to come to an understanding of such processes both experimentally and in theory^12–15^.

When experimentally studying the evolution of dissipative structures of active Brownian grains, it is necessary to find the driving force that can be controlled during the experiment. For active Brownian grains, such force can be associated with the mechanism of their active motion and therefore determine the intensity of such motion (kinetic energy and diffusion coefficient of grains). Thus, a controlled change in force can influence the evolution of a system of active Brownian grains.

One of the mechanisms of active motion of Brownian grains is photophoresis^16^. As soon as a grain in the gas is illuminated, the absorption, diffraction, refraction and reflection of light by the grain occur along with the release of heat on the grain surface. The action of laser radiation on the grain can lead to the appearance of a photophoretic (radiometric) force^16,17^ associated with the nonuniform heating of the single grain surface by the laser beam. When the grain size is much larger than the wavelength of laser radiation, this is due to inhomogeneous illumination of the surface of a single spherical grain (in the geometrical optics approximation)^16,18–20^. As a result, a temperature gradient arises so that collisions of gas molecules with the heated surface of the grain lead to the radiometric force.

The aim of this work is to study the Brownian motion of light-absorbing and strongly interacting grains in a quasi-two-dimensional system (monolayer) far from equilibrium under conditions when the nature and intensity of Brownian motion depend on the effect of laser radiation. In the experiment, metal-coated polymer grains levitate in gas discharge. At this point, the absorption of laser radiation by the metal-coated surface of the grain creates a radiometric force, which in turn drives the grain. This paper presents the results of observations confirming the active nature of the Brownian motion of such grains. We also study the change in the nature of the Brownian motion of grains at different time scales and the change in the intensity of motion of grains (their kinetic energy and diffusion) with varying laser radiation power.

## Experiments

**Figure 1:**
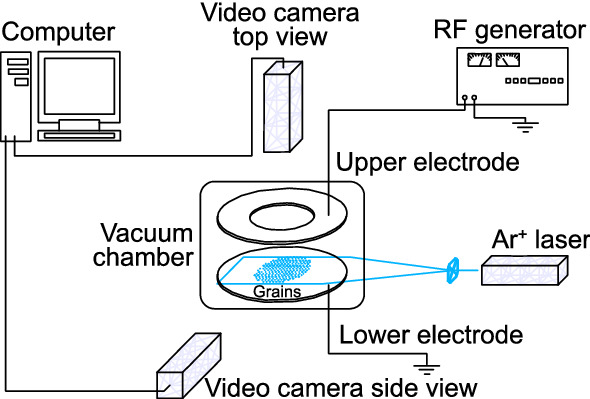
Scheme of the experimental setup.

The experiments are performed in an RF discharge in argon at pressure *P* = 3 Pa, with a discharge power *W* = 13 W. The grains are melamine–formaldehyde of density *ρ*_*p*_ = 1.51 g·cm^−3^, coated with copper of 200 nm in thickness, with a diameter *d*_*p*_ of 9.95 μm. The scheme of the experimental setup is presented in Fig. 1.

The grains after injection in the discharge volume gain negative charges because of ion and electron fluxes going to the grain surface until a stationary state is reached. Due to the balance of gravity and electrostatic force, providing stiff vertical confinement, charged grains levitate above a flat horizontal RF electrode, forming a monolayer structure with negligible small vertical motions. To prevent the escape of grains in the horizontal direction, metal rings with heights of approximately 3 mm and 60 mm in diameter are mounted on the lower (grounded) electrode; the ring forms a potential trap for the cloud of grains.

Grains gain sufficiently high electrical charge (~ 10^2^–10^5^ electron charges) under the flow of plasma particles^21,22^. These charged grains effectively interact with each other as well as with an external electrical field. The action of external forces and forces of interaction combined with dissipative mechanisms in these systems can lead to the self-organization of the system, resulting in the formation of quasistationary crystal- or liquid-like structures^21–33^.

An expanded laser beam is formed by a plano-cylindrical lens with a cross section of 7 × 120 mm. The distribution of the beam intensity in the region of interaction with the monolayer structure of dust grains is substantially uniform (the homogeneity region is 1 × 60 mm). The wavelength of the laser radiation is 514.5 nm. The observed grain cloud consists of a single layer with a diameter of 32 mm and a number of grains of approximately 1500. This monolayer is videotaped using a high-speed videocamera (frame frequency 200 s^−1^).

The laser power varies in the range from 20 to 140 mW, measured before the grain. The method of kinetic heating of grains in structures, under constant plasma parameters of the discharge, is based on the influence of an enlarged homogeneous laser beam on the entire grain system. The grains acquire kinetic energy of chaotic motion due to the action of radiometric forces^16^, resulting in melting of the crystal-like grain monolayer.

For visualization, the laser radiation scattered by the grains is recorded by a video camera. The resulting video recording of grains in the structure is processed by the following software algorithm. First, static noise in the video image is detected and removed, and low- and high-frequency spatial noise is filtered. Next, the search for local maxima in the filtered image is performed. For each maximum, the weighted average coordinate and the integrated brightness of circles considered as grains are determined, and images with too low brightness are deleted. Next, the search for grains on all frames of the video (to restore their trajectory) is carried out using the maximum likelihood method. The likelihood is calculated from the relative displacements of a grain from frame to frame and its brightness, taking into account the behavior of neighboring grains. Finally, the heuristic algorithm processes special cases (intersections and discontinuities of trajectories) and determines the final set of grain trajectories.

The analysis of the obtained video data makes it possible to determine the coordinates of individual grains at each moment of time, while the analysis of the displacements of grains for the interframe interval gives the speed ${v}_{p}$ of their movement. Based on the data about the velocities of all grains of a dusty system at each moment of time, it is possible to obtain the velocity distribution of grains and their average kinetic energy of grain motion ${E}_{k}={m}_{p}{v}_{p}^{2}/2$.

Quantitative and qualitative analysis of the state of a system is performed as a rule using pair *g*(*r*) correlation functions^21–31,34–37^. Recall that the pair function *g*(*r*) determines the probability of finding two grains separated by a distance *r* and is a measure of the translational order in a system of interacting grains; *g*(*r*) = 1 for ideal gases.

In general, the pair correlation function is defined as *g*(*r*) = (1/*n*_*p*_)(*dN*(*r*)/*dV*(*r*)) and is calculated by sequentially choosing each grain as the central grain while counting the number of grains d*N*(*r*) located at distance *r* from the selected grain on a ring with area d*V*(*r*) = 2π*r*d*r* (for two-dimensional systems). The distance *r* varies from zero to *r*_max_, which is equal to the maximum possible radius of a circle that can be inscribed in the investigated region of the system centered at the selected grain. Then, the obtained results are averaged over the ensemble and time (frames) and normalized to the average grain density *n*_*p*_.

For diagnostics of grain parameters, the effective coupling parameter

1 $$\Gamma^{*} = {1}.{5}\left( {eZ} \right)^{{2}} ({1} + \kappa + \kappa^{{2}} /{2}){\text{exp}}( - \kappa )/(T_{eff} r_{p} )$$

and scaling parameter

2 $$\xi = \left( {\omega^{*} /\nu_{fr} } \right)$$

where.

3 $$\omega^{*} = \left\{ {{2}\left( {eZ} \right)^{{2}} \left( {{1} + \kappa + \kappa^{{2}} /{2}} \right){\text{exp}}( - \kappa )/\left( {r_{p}^{{3}} \pi m_{p} } \right)} \right\}^{{{1}/{2}}}$$

the technique based on analysis of the mass transfer processes for a small observation time^31,38^ is used. Here, *κ*≡*r*_*p*_/λ is the screening parameter, ${k}_{B}$ is Boltzmann's constant, λ is the screening length, *r*_*p*_ is the intergrain distance, *Z* is grain electric charge, *T*_*eff*_ is the kinetic (effective) temperature of the grains, ${m}_{p}$ is mass of the grain and *ν*_*fr*_ is a coefficient of the friction of grains due to their collisions with neutrals of the ambient gas.

Under experimental conditions, the Γ^*^ values vary from 10 to 525, the mean intergrain distances *r*_*p*_ are approximately 800 μm, the characteristic frequency ω^*^≈ 9.9 ± 2 and the friction coefficient*ν*_*fr*_≈1.7 s^−1^.

## Results and discussion

The action of laser radiation on the grain can lead to the appearance of a photophoretic force associated with the inhomogeneous illumination of the single grain surface by the laser beam and, as a result, the nonuniform heating of single grains and an uneven temperature distribution over the grain surface when the grain size is much larger than the wavelength of laser radiation (in the geometrical optics approximation for a single spherical grain). In this case the force is called radiometric^16,18–20^.

The phenomenon leading to the radiometric effect has a molecular-kinetic origin: gas molecules colliding with the warmer side of the grain surface after bouncing back have a higher average kinetic energy than molecules colliding with the less heated side of the surface. This means that molecules, when reflected from the warmer side of the grain, give more momentum to it than molecules reflected from the less heated side. Thus, spontaneous symmetry breaking arises^39^, while an uncompensated impulse is transmitted to the grain, fluctuating in magnitude and direction. As a result, the intensity of Brownian motion of the grain, both translational and rotational, increases^16,17,19,20,40^.

**Figure 2:**
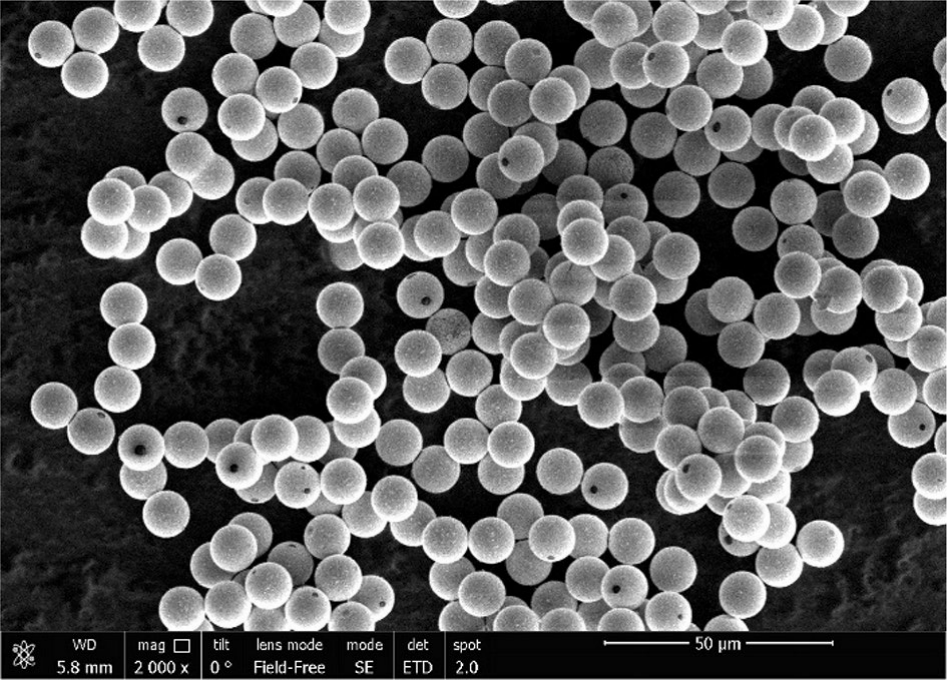
Scanning electron microscopy photograph of a metal-coated grain.

Figure 2 shows an image of the copper-coated grains (manufacturer—microParticles GmbH) obtained by scanning electron microscopy. Almost the entire surface of grains is covered with metal; however, there may be small coating defects on the surface that violate its continuity. No more than 20% of grains have a visible defect (on scanning electron microscopy photograph), while the defect area is less than 1% of the grain surface area.

Let us estimate the change in the temperature of grains upon absorption of laser radiation for times δt∼1 s, which characterizes the performing of a stationary state of a dusty plasma structure under the action of laser radiation with a power of 0.1 W. The area of the laser beam in the region of the structure is 0.175 × 0.005 m^2^ = 8.75⋅10^−4^ m^2^. The incident power density of the laser radiation is approximately 1.1⋅10^2^ W/m^2^. The area for evaluating the absorption of a spherical grain ∼π*d*_*p*_^2^/4 (*d*_*p*_ ∼10⋅10^−6^ м) is approximately 7.85⋅10^−11^ m^2^. The power of laser radiation reaching the grain is approximately 9.0⋅10^−9^ W. The absorption coefficient of radiation in the visible spectrum for copper is 0.5^41^. Consequently, the power of the laser radiation absorbed by the dust grain is approximately 4.5⋅10^−9^ W. The volume of a dust grain π*d*_*p*_^3^/6 is approximately 5⋅10^−16^ m^3^. The density of melamine formaldehyde is 1.5⋅10^3^ kg/m^3^. The mass *m*_*p*_ of a melamine formaldehyde grain is approximately 7.5⋅10^−13^ kg, and the mass of copper *m*_Cu_ per grain is approximately 5.4⋅10^−13^ kg. The heat capacity of copper C_Cu_ is 381 J/kg·K^41^. The heat capacity of melamine formaldehyde C_MF_ is 1000 J/kg·K^42^. Accordingly, laser radiation with a power of 0.1 W can lead to heating of dust grains with a speed δT/δt = Q/(C_Cu_m_Cu_ + C_MF_m_MF_) ∼ 4–5 К/c.

**Figure 3:**
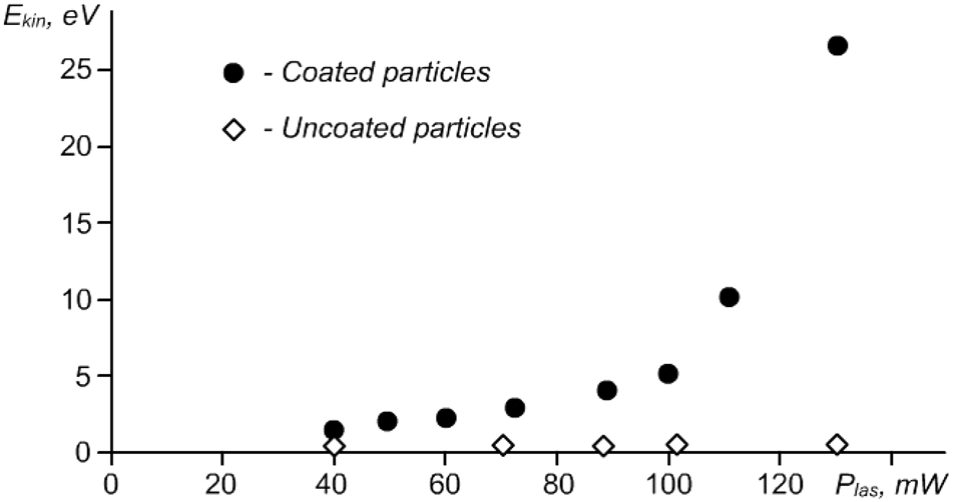
Kinetic energy *E*_*kin*_ of uncoated melamine–formaldehyde (rhombus) and copper-coated melamine–formaldehyde (circles) grains vs the power *P*_*las*_ of external laser radiation. The diameter of all grains is 9.95 μm, the pressure of buffer gas (argon) is 3 Pa, and the power of discharge is 13 W.

The measured kinetic energy *E*_*k*_ of grains as a function of laser power is shown in Fig. 3. All data on the kinetic temperature of grains are given for a stationary state under stationary laser heating. We compared the motion of uncovered melamine–formaldehyde (MF) grains in RF discharge and the same MF grains covered with a thin layer of copper. The difference in the kinetic energy *E*_*kin*_ is dramatic (see Fig. 3). It is easy to see that the kinetic energy of metal-coated grains is essentially higher than that of uncovered grains and directly depends on the intensity of the radiation (power *P*_*las*_ of the laser).

As seen from the figure, with an increase in the power *P*_*las*_ of laser radiation by approximately 3.5 times, the kinetic energy ${E}_{kin}={m}_{p}{v}_{p}^{2}/2$ of the observed grains (grains within the area exposed by the laser beam) increases by approximately 20 times. This means that there is a mechanism for converting the energy of an external source (laser radiation) into the energy of their kinetic motion, i.e. they are open systems, and the motion of the grains themselves can be characterized as the motion of active Brownian grains^1^ with strong Coulomb interactions.

With an increase in the kinetic energy of grains, the transition of the grain monolayer from the crystal to liquid state is observed.

**Figure 4:**
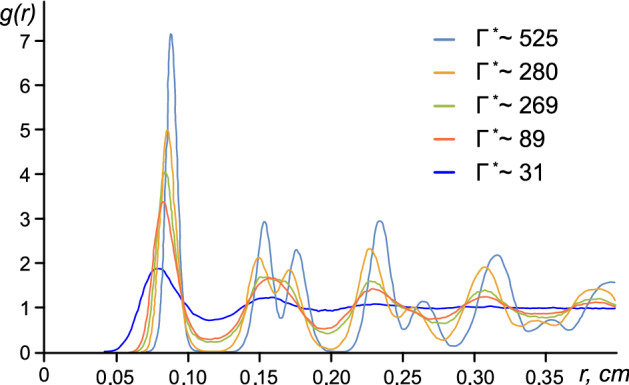
Pair correlation functions *g*(*r*) for different states of grain monolayers, solid and liquid, at different values of the coupling parameter, Γ^*^: 1—Γ^*^ = 525 (solid); 2—Γ^*^ = 280 (solid); 3—Γ^*^ = 269 (liquid); 4—Γ^*^ = 89 (liquid); 5 – Γ^*^ = 31 (liquid).

Figure 4 summarizes the behavior of the system at different Γ^*^ parameter values near the melting transition. The pair correlation functions *g*(*r*) for different states of grain monolayer, solid and liquid are shown.

**Figure 5:**
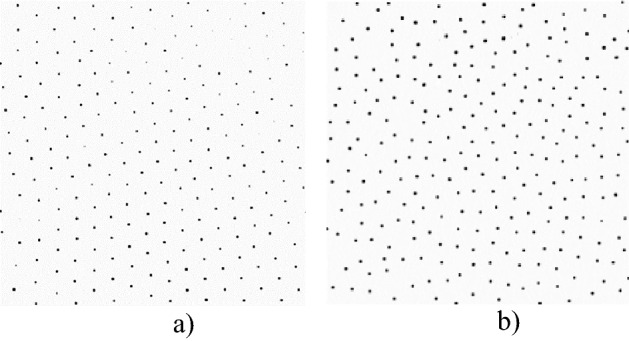
Single layer of the observed grain cloud at different values of laser power *P*_*las*_, effective coupling parameter Γ^*^ and kinetic (effective) temperature of the grains: (**a**) Γ^*^ = 525, *P*_*las*_ = 40 mW, *E*_*kin*_ = 1.38 eV; (**b**) Γ^*^ = 31, *P*_*las*_ = 131 mW, *E*_*kin*_ = 26.7 eV.

Typical images of a monolayer of the grains at different values of laser power *P*_*las*_, effective coupling parameter Γ^*^ and kinetic energy of the grains are shown in Fig. 5 (see also Supplementary Video file “V1.mp4”). There is a clearly visible change in the structure of the monolayer with the growth of *P*_*las*_.

**Figure 6:**
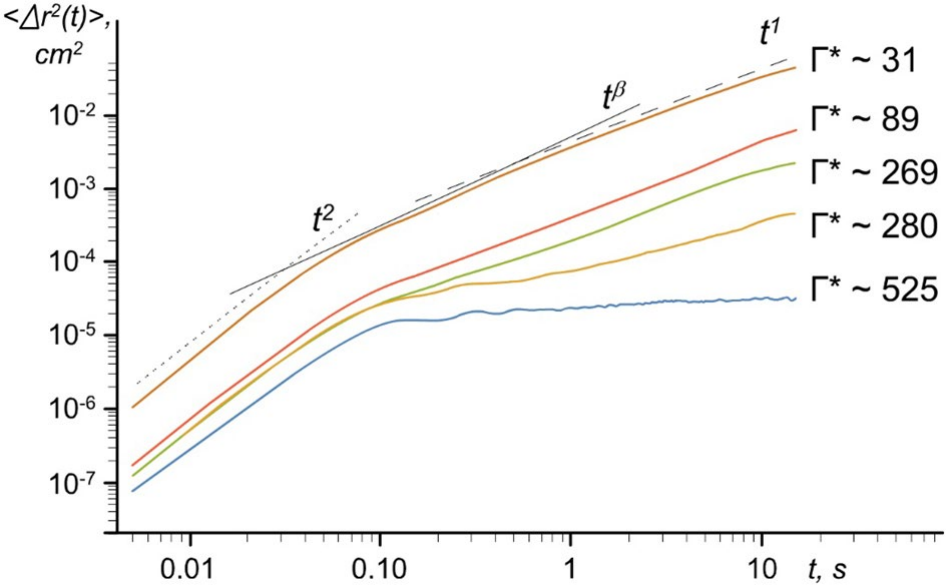
The mean square displacement (MSD) < Δ*r*^2^(*t*)) > of the moving grains for different states of grain monolayer, solid and liquid, at different values of the coupling parameter, Γ^*^: 1—Γ^*^ = 525; 2—Γ^*^ = 280; 3—Γ^*^ = 269; 4—Γ^*^ = 89; 5—Γ^*^ = 31.

In our experiments, we measure the mean square displacement (MSD) < *r*^2^(*t*)) > of the moving grains. The MSDs for different laser irradiation power *P*_*las*_ are shown in Fig. 6, and the corresponding values of the measured diffusion coefficient *D*_*p*_ lie in the range from ~ 10^–5^ cm^2^/s to ~ 4⋅10^–3^ cm^2^/s.

Such grains differ from passive Brownian grains^2^, in whh the mechanism of grain motion is associated with thermal fluctuations in the surrounding fluid, and the diffusion coefficient ${D}_{p}^{o}$ is determined by the Einstein formula^43^:

4 $$D_{p}^{o} = \frac{{k_{B} T_{Ar} }}{{6\pi \left( {d_{p} /2} \right)\nu_{n} }}$$

where *T*_*Ar*_ is the temperature of the gas and ${\nu }_{n}$ is the viscosity of the gas.

In our experiments with laboratory plasmas, argon at pressure *P* = 3 Pa is used as a buffer gas. In this case, the coefficient of dynamic viscosity can be calculated using the Epstein formula^44^:

5 $$\nu_{n} = \delta \frac{16}{{9\pi }} \frac{P}{{u_{n} }} \left( {d_{p} /2} \right)$$

where *δ* = 0.5 + π/8 for specular reflection of neutrals from the grain surface, δ = 1 + π/8 for complete accommodation^45,46^, and *u*_*n*_ is the average thermal velocity of neutrals.

From Einstein's formula (4), the following value of the diffusion coefficient can be obtained *ν*_*n*_ = 1.29⋅10^–6^ Pa‧s and ${D}_{p}^{o}$ ≈ 2.2⋅10^–5^ cm^2^/s (*T*_*Ar*_≈300 K), which is comparable (at Γ^*^ = 525) or less (at Γ^*^ = 31) than those obtained from experiments, by 2 orders of magnitude (hot Brownian motion^47^).

We observe that laser irradiation increased the MSD (and therefore decreased the coupling of charged grains), and we also see the confining of the grain for an extended period of time *τ* (see Fig. 6, curves at Γ^*^ = 525 and 280, *τ* > 0.1 s, corresponding to the solid state). The latter can be explained by the two-dimensional trapping effect due to a strong grain Coulomb interaction^48,49^.

At a low intensity of laser radiation (coupling parameter 525, which corresponds to the solid state), the grains are trapped by energy barriers (due to Coulomb interaction). An increase in radiation intensity leads to an increase in the kinetic energy of the grains and a decrease in energy to overcome the Coulomb energy barrier. In addition, the grains can move freely (Fig. 6, curves at Γ^*^ = 269, 89 and 31, corresponding to the liquid state). It should be noted that at shorter times (*τ* < 0.01 s), the motion of grains is ballistic (< Δ*r*^2^(*t*)) >  ~ *t*^*2*^). For long times (0.5 < *τ* < 10 s), however, the data are consistent with normal diffusion, with < Δ*r*^2^(*t*)) >  ~ *t*.

For short times (0.1 < *τ* < 0.5 s) a transition (crossover) from the ballistic regime to the diffusion regime is observed. Note that we cannot make an unambiguous conclusion about the nature of the transition. Thus, possible mechanisms affecting the mode of the transition from the ballistic regime to the diffusion regime were considered in a number of works^52,53^, where the transition from the ballistic regime to diffusion was detected for a paradigmatic model of nonequilibrium statistical physics. Perhaps, the grain diffusion can be considered anomalous (or superdiffusive) (Fig. 6, curve at Γ^*^ = 31) with < Δ*r*^2^(*t*)) >  ~ *t*^*β*^ , where 3/2 < *β* < 2 for short times (0.1 < *τ* < 0.5 s) and <  > represents the ensemble average^50,51^.

In our experiments, the duration of continuous video recording of particles was limited to 4000 frames, which was 20 s at a video recording speed of 200 fps. Video recording time was limited by the hardware capabilities of a high-speed video camera (its memory buffer). Thus, we could not observe the dependence MSD on *t* at long enough times, when a final crossover to diffusive behavior can appear, for instance, in the frame of mobile immobile transport models for exponential trapping time densities^54^, with Poissonian switching^55^, and in stochastic approach addressed, in particular, to systems with non-Gaussian, anomalous diffusion^56^.

Based on these observations, we consider that the dynamics of the copper-coated grains change at different laser powers through trapping confinement (Fig. 6, curves at Γ^*^ = 525 and 280), hot Brownian motion (Fig. 6, curves at Γ^*^ = 269, 89 and 31) and crossover for short times (0.1 < *τ* < 0.5 s) (Fig. 6, curve at Γ^*^ = 31).

It is advisable to analyze the differences between passive Brownian motion and active Brownian motion, as introduced in reference^1^, considering the average grain trajectory over the ensemble, given by the initial position and orientation of the motion at time *t* = 0, i.e., *x*(0) = *y*(0) = 0, where (x, y) is the position of the grain. For passive Brownian motion, this averaging, for reasons of symmetry, leads to a value equal to zero, i.e., < *x*(*t*) >  =  < *y*(*t*) >  = 0, where <  > is the ensemble mean.

If we consider the active motion of grains, then we can expect that, as a result of averaging, we will obtain a straight line along the *x* direction determined by a given initial orientation (< *x*(*t*) > , with < *y*(*t*) >  = 0 due to symmetry^1^. This means that, on average, the active Brownian grain will move along the direction of its initial orientation at some finite length *L* (persistence length^1^) before its motion becomes random.

**Figure 7:**
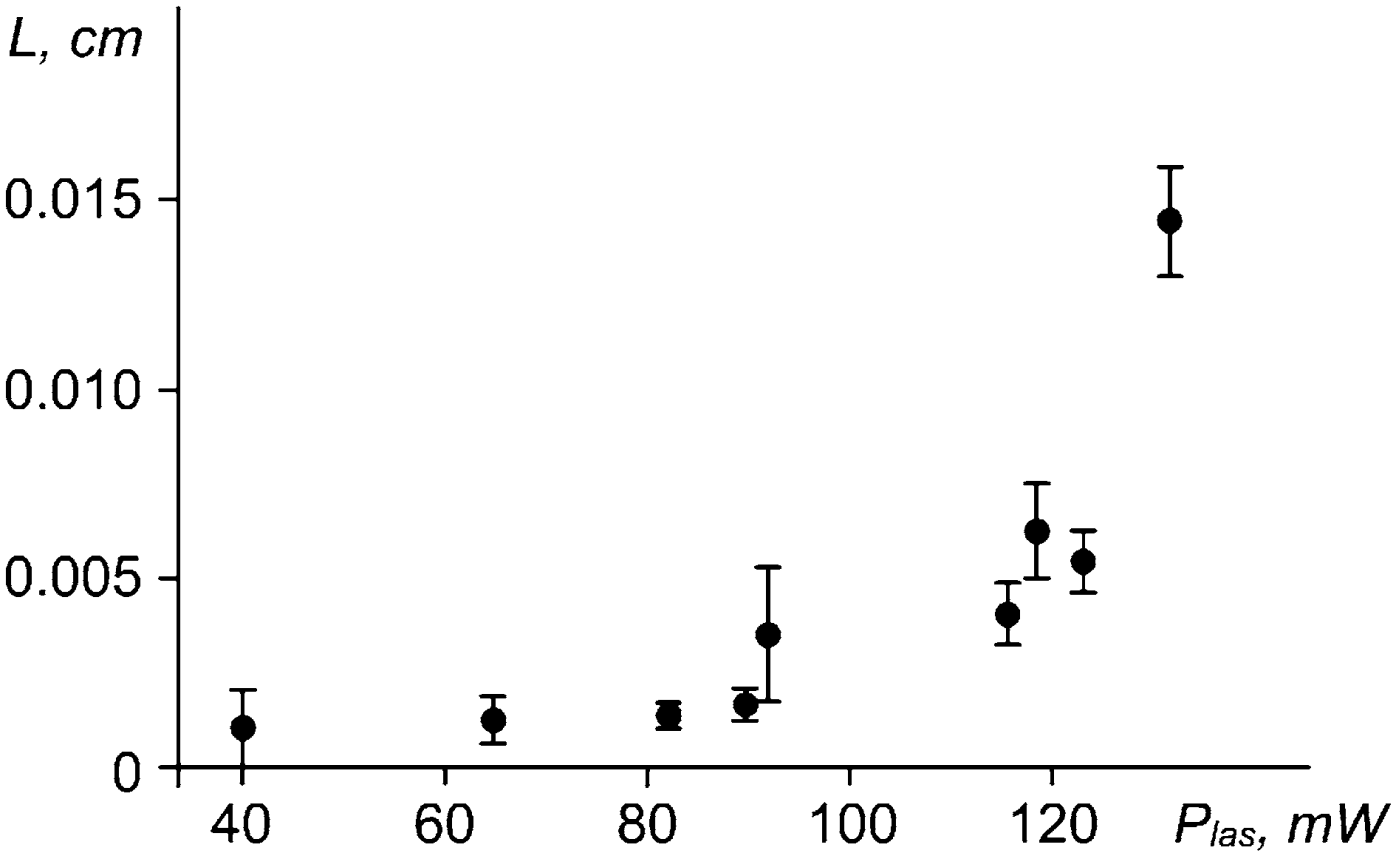
Dependence of the persistence length *L* of the moving grains on the laser power *P*_*las*_.

**Figure 8:**
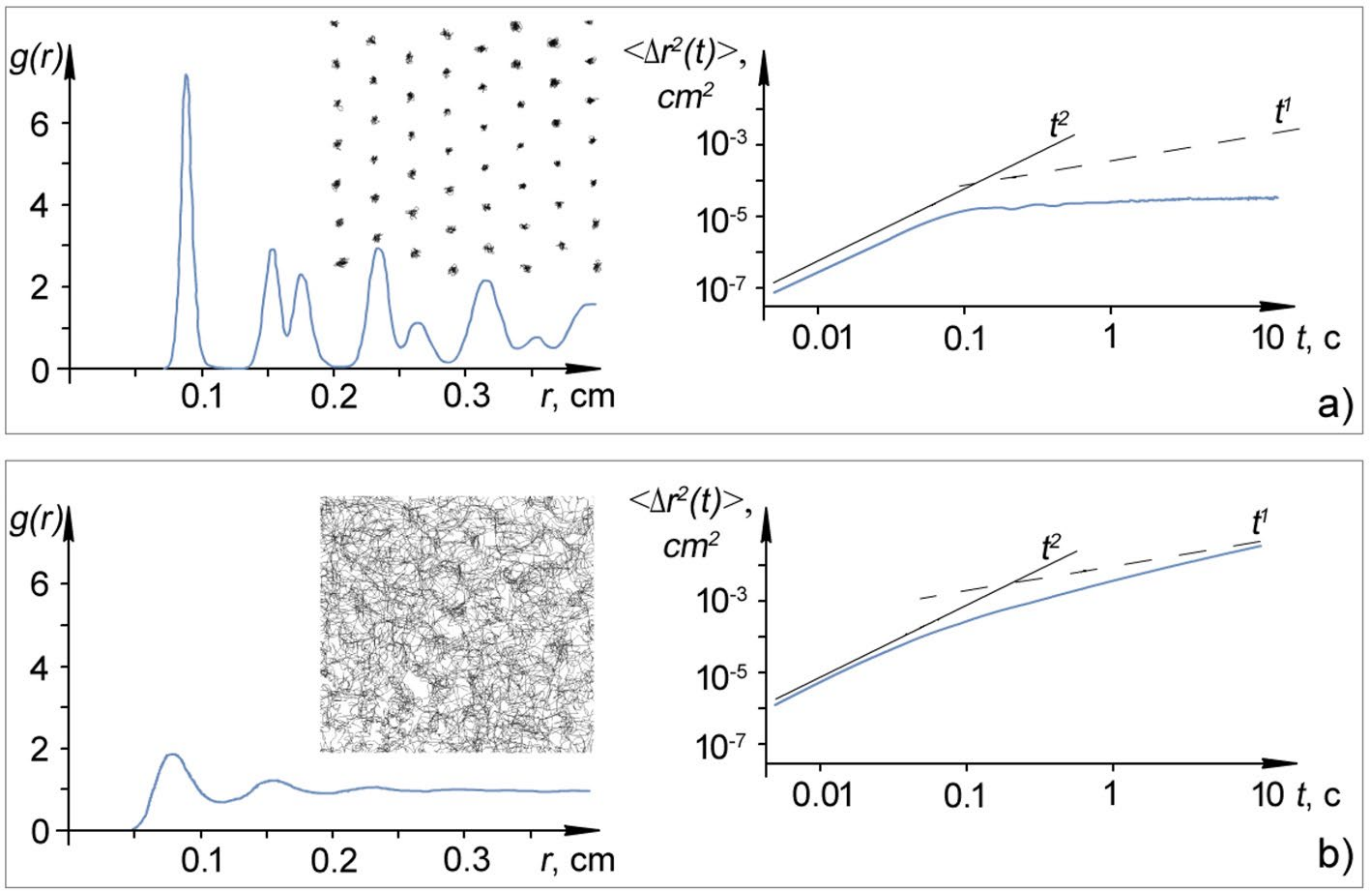
Pair correlation functions and root mean square displacements of grains and persistence length *L* at different states of the grain monolayer characterized by different values of laser power *P*_*las*_ and kinetic energy *E*_*kin*_: (**a**) Γ^*^ = 525, *P*_*las*_ = 40 mW, *E*_*kin*_ = 1.38 eV, *L* = 0.00107 cm; and (**b**) Γ^*^ = 31, *P*_*las*_ = 131 mW, *E*_*kin*_ = 26.7 eV, *L* = 0.0148 cm. The insets to the graphs show the trajectories of grains in the structure during t = 5 s for different values of coupling parameter Г^*^, average kinetic energy *E*_*kin*_ and persistence length *L.*

We define the persistence length *L* as < *x*(*t*) > for long times (0.5 < *τ* < 10 s), considering the averaging over the ensemble. The results for *L* with different values of the laser power *P*_*las*_ are shown in Fig. 7 and Fig. 8a, b. Additionally, in Fig. 8, there are pair correlation functions, two-dimensional trajectories of a grain and MSD < *r*^2^(*t*)) > of grains. The increase in persistence length with increasing laser power also indicates that grains become more active. This is consistent with the results of the MSD measurements discussed above.

Also, we should note, that there are fundamental differences between the nature of active Brownian motion in rarefied media, such as plasma-forming gas in RF and DC discharges at low pressures, and motion in dense media, such as liquids. In particular, in rarefied gas media, including weakly ionized plasmas, both overdamped and underdamped motions are possible^57^.

## Conclusion

We present the results of the study of the Brownian motion of metal-coated charge grains far from thermodynamic equilibrium suspended in gas discharge during the melting of their monolayer under laser action. The motion is caused by photophoresis, i.e. absorption of laser radiation at the metal-coated surface of the grain creates radiometric force, which in turn drives the grain. We experimentally observe the active Brownian motion of strongly coupled charged grains under different laser powers. The radiometric force can lead to an effective increase in the kinetic energy and the diffusion coefficient of spherical grains by several orders of magnitude in comparison with passive Brownian motion.

## Supplementary Information

Supplementary Video 1.
